# The value of desmosomal plaque-related markers to distinguish squamous cell carcinoma and adenocarcinoma of the lung

**DOI:** 10.1080/03009734.2019.1692101

**Published:** 2019-12-06

**Authors:** Inmaculada Galindo, Mercedes Gómez-Morales, Inés Díaz-Cano, Álvaro Andrades, Mercedes Caba-Molina, María Teresa Miranda-León, Pedro Pablo Medina, Joel Martín-Padron, María Esther Fárez-Vidal

**Affiliations:** a Department of Pathology, School of Medicine, University of Granada, Granada, Spain;; b Department of Biochemistry and Molecular Biology III, School of Medicine, University of Granada, Granada, Spain;; c Centre for Genomics and Oncological Research (GENYO), Granada, Spain;; d Department of Biochemistry and Molecular Biology I, University of Granada, Granada, Spain;; e Department of Statistics and Operative Research, School of Medicine, University of Granada, Granada, Spain;; f Institute for Biomedical Research (IBS Granada), University Hospital Complex of Granada/University of Granada, Granada, Spain

**Keywords:** Adenocarcinoma, desmosomal plaque proteins, non-small-cell lung cancer, squamous cell carcinoma

## Abstract

**Background:** An antibody panel is needed to definitively differentiate between adenocarcinoma (AC) and squamous cell carcinoma (SCC) in order to meet more stringent requirements for the histologic classification of lung cancers. Staining of desmosomal plaque-related proteins may be useful in the diagnosis of lung SCC.

**Materials and methods:** We compared the usefulness of six conventional (CK5/6, p40, p63, CK7, TTF1, and Napsin A) and three novel (PKP1, KRT15, and DSG3) markers to distinguish between lung SCC and AC in 85 small biopsy specimens (41 ACs and 44 SCCs). Correlations were examined between expression of the markers and patients’ histologic and clinical data.

**Results:** The specificity for SCC of membrane staining for PKP1, KRT15, and DSG3 was 97.4%, 94.6%, and 100%, respectively, and it was 100% when the markers were used together and in combination with the conventional markers (AUCs of 0.7619 for Panel 1 SCC, 0.7375 for Panel 2 SCC, 0.8552 for Panel 1 AC, and 0.8088 for Panel 2 AC). In a stepwise multivariate logistic regression model, the combination of CK5/6, p63, and PKP1 in membrane was the optimal panel to differentiate between SCC and AC, with a percentage correct classification of 96.2% overall (94.6% of ACs and 97.6% of SCCs). PKP1 and DSG3 are related to the prognosis.

**Conclusions:** PKP1, KRT15, and DSG3 are highly specific for SCC, but they were more useful to differentiate between SCC and AC when used together and in combination with conventional markers. PKP1 and DSG3 expressions may have prognostic value.

## Introduction

Lung cancer is one of the most widely prevalent cancers and has the highest cancer-related mortality rate worldwide (1), with small-cell lung cancer (SCLC; ≈15%) and non-small-cell lung cancer (NSCLC; ≈85%) being the two main histological types. NSCLC can be divided into three major histological subtypes: squamous cell carcinoma (SCC), adenocarcinoma (AC), and large-cell lung cancer. AC and SCC account for the vast majority of NSCLC cases. SCCs usually arise in a main or lobar bronchus and are therefore more frequently localized centrally in the lung, while the most common localization of invasive AC is the lung periphery (2). Accurate histological subclassification of NSCLCs is crucial, because the therapeutic approach depends on their histological type. Therefore, the latest revision of the World Health Organization (WHO) classification of lung neoplasms includes guidelines for the terminology and procedure to be followed in order to classify pulmonary neoplasms in small biopsies, recommending immunohistochemical analysis when available (2,3). For instance, epidermal growth factor receptor (EGFR) inhibitors and the anti-folate agent pemetrexed are recommended for the treatment of AC but not SCC (4,5). Moreover, EGFR mutations and gene fusions including ALK and ROS1 are almost exclusively present in non-squamous cancer forms. Further, patients with AC who possess the fusion gene *EML4–ALK* (echinoderm microtubule-associated protein-like 4*–*anaplastic lymphoma kinase) or *EGFR* gene-activating mutations can respond to the respective tyrosine–kinase inhibitors (6,7). Additionally, SCC patients should not be treated with the anti-vascular endothelial growth factor agent bevacizumab, which frequently produces lung haemorrhage (8). The identification of new therapeutic targets means that tissue samples are used not only for diagnosis but also for immunohistochemical staining and molecular testing in relation to potential therapy (3). This is particularly challenging when small biopsies or cytology smears are the only material available, as in 70% of lung cancer patients with advanced disease and inoperable neoplasms at diagnosis (3). These challenges led to new classification proposals for non-resection specimens, biopsies, and cytology, including the ASLC/ATS/ERS lung adenocarcinoma classification and the latest revision of the WHO lung cancer classification, which include the need for ancillary techniques such as immunohistochemistry (2,9). With the application of these techniques, the accurate diagnosis of AC or SCC can improve from 50–70% to above 90% (10,11). The search for novel markers to accurately differentiate between AC and SCC is therefore of major clinical relevance.

Desmosomes are cell structures specialized for focal cell-to-cell adhesion that are localized in randomly arranged spots on the lateral sides of plasma membranes. They play an important role in providing strength to tissues under mechanical stress, such as the cardiac muscle and epidermis. Besides the constitutive desmosomal plaque proteins desmoplakin and plakoglobin, at least one of the three classical members of the plakophilin (PKP) family is required to form functional desmosomes (12–14). PKP1 is a major desmosomal plaque component that recruits intermediate filaments to sites of cell–cell contact via interaction with desmoplakin. PKPs regulate cellular processes, including protein synthesis and cell growth, proliferation, and migration, and they have been implicated in tumour development (15–21).

Desmoglein 3 (DSG3) is one of seven desmosomal cadherins. Desmosomal proteins act as tumour suppressors and are downregulated in epithelial–mesenchymal transition and in tumour cell invasion and metastasis. However, some studies have shown the upregulation of several desmosomal components in cancer, including DSG3, and overexpression of these proteins has been related to the prognosis. Therefore, desmosomal proteins can potentially serve as diagnostic and prognostic markers (22). Keratin 15 (KRT15) is a type I keratin protein present in the basal keratinocytes of stratified epithelium. For this reason, it has been reported as a marker of stem cells. However, several studies have demonstrated KRT15 expression in differentiated cells (23). Our group previously reported that gene sequences corresponding to the desmosomal plaque-related proteins PKP1, DSG3, and KRT15 were differentially expressed in primary AC and SCC of the lung (24). Subsequently, we also described the localization of PKP1 in nucleus, cytoplasm, and cell membrane in tumours and proposed the utilization of these proteins as immunohistochemical markers (25).

Immunohistochemistry is widely used for the subtyping of lung carcinomas. Thyroid transcription factor 1 (TTF1) (26) and Napsin A (27) are considered the most useful markers for AC diagnosis, and evaluation of the former is considered easier because it is a nuclear marker. Although cytokeratin 7 (CK7) has also been used as a marker of AC (28), its usefulness is not universally accepted (2). Cytokeratin 5/6 (CK5/6), p63, and p40 are recommended markers for SCC (28,29), while DSG3 and desmocollin 3 have also emerged as potential SCC markers, although their clinical value has yet to be established (25,30,31). However, despite the efficacy of these markers, numerous confirmed lung carcinoma cases are either positive for both AC and SCC markers (double-positive) or negative for one or the other type of marker (32). Given the more stringent requirements for the histologic classification of lung cancers, an antibody panel is required that definitively differentiates AC from SCC. A particular challenge is posed by poorly differentiated tumours and by samples with the technical artefacts frequently encountered in small biopsy specimens, which are the only available tissue samples from patients in advanced stages.

In this study, we compared the usefulness of six conventional and three novel markers for the differential diagnosis of lung SCC and AC in small biopsy specimens. We also explored correlations between the expression of these markers and the histologic and clinical data of the patients.

## Materials and methods

### Tumour tissues

Specimens used in this study were from 87 patients who underwent surgical resection for lung cancer. All patients were stages I (54 patients), II (20 patients), or III (10 patients), except for three patients in stage IV, and were selected for surgery with no previous chemotherapy or radiotherapy treatments. Only cases in which the histological diagnosis of the resected specimen was invasive AC or SCC were included, representing the majority of the series (85 patients). The remaining two cases, which corresponded to sarcomatoid carcinoma, were excluded from the study.

Histological diagnosis of the surgical specimens followed the recommendations of the WHO 2015 classification (2), based on morphology and ancillary techniques, mainly immunohistochemistry, when needed. Therefore, the present study included 85 samples from primary malignant lung neoplasms (41 ACs and 44 SCCs) in stage I (54 patients), stage II (18 patients), stage III (10 patients), or stage IV (3 patients), which were obtained by incisional biopsy of ≤0.4 cm during tumour surgery. Samples were divided into two, and one half was used for RNA extraction, the results of which have previously been published (24). The other half was fixed in buffered formalin and embedded in paraffin blocks, from which 3–4-μm sections were cut for conventional haematoxylin and eosin staining.

The 85 incisional biopsies were analyzed by two pathologists (authors MGM and MCM) and classified according to the WHO 2015 classification (2), cataloguing those with keratinization and/or intercellular bridges as SCCs and those with glandular differentiation and/or mucin production as ACs, including acinar, papillary, lepidic, or solid growth patterns. In addition, SCCs were classified as well-differentiated if keratinization and/or intercellular bridges were observed in more than 75% of the tumour sample, moderately differentiated if observed in 25–75%, and poorly differentiated if observed in less than 25%. Likewise, ACs were subclassified as well-differentiated when glandules were formed in more than 75% of the tumour sample, moderately differentiated when formed in 25–75%, and poorly differentiated when observed in less than 25%. Diagnoses of the biopsy samples were compared with the definitive diagnoses of the surgical specimens.

Before the study, all medical records and tumour sections from surgical specimens were reviewed. Informed consent was obtained from all participants. The study protocol complied with the Helsinki Declaration of 1975 as revised in 1983. Written informed consent was obtained from all patients for the study, which was approved by the Ethics Committee of our Institution (Clinical Trial Committee of San Cecilio University Hospital, Granada). Pathological evaluation of resected specimens was carried out using the 1997 revision of the International System for Staging Lung Cancer (33) and revised according to the most recent (7th) edition of the tumour, node, and metastasis classification of the International Union Against Cancer (UICC) staging system. All specimens were primary tumours with no history of treatment that could affect the immunohistochemical results.

### Immunohistochemistry

Sections of 4-μm thickness were taken from the paraffin blocks, mounted on pre-treated slices, and stained for PKP1 (rabbit polyclonal, HPA027221; Sigma, St Louis, MO, USA), KRT15 (rabbit polyclonal, HPA024554; Sigma), and DSG3 (mouse monoclonal, NB100-1643; Abcam, Cambridge, UK). Normal human skin was used as positive control. After testing several dilutions, PKP1 and DSG3 were diluted at 1:200 and KRT15 at 1:500. Prediluted monoclonal antibodies from Master Diagnostica were used for the remaining determinations: TTF1 (000486QD, clone SPT24), Napsin A (001004QD, clone BS10), CK7 (001004QD, clone OVTL 12/30), CK5/6 (000680QD, clone EP24/EP67/B22-18B231), p63 (000479QD clone 4^a^4), and p40 (000686QD, clone ZR8).

Normal lung tissue was used as a positive control for TTF1 and Napsin A, breast cancer for CK7, and skin SCC for CK5/6, p63, and p40. As negative controls, the same positive controls were used, replacing the primary antibody with PBS.

Immunohistochemical staining was carried out by automatic immunostaining (LabVision Autostainer 480 s Thermofisher) using the Master Polymer Plus Detection System (MAD-000327QK Master Diagnostic) and following the manufacturer’s instructions. Diaminobenzidine was used as chromogen, and sections were counterstained with haematoxylin. Immunohistochemical staining was evaluated independently by two pathologists (authors MGM and MCM), who assigned a score based on the extent and intensity of immunoreactivity. Agreement between pathologists was >90%, and any differences in interpreting results were resolved by consensus. Staining for PKP1, CK15, and DSG3 was evaluated semiquantitatively in nucleus, cytoplasm, and membrane as negative (0, <5% cells stained), positive 1+ (6–25% cells stained), positive 2+ (26–50% cells stained), or positive 3+ (>50% cells stained). Staining intensity at the same localizations was scored semiquantitatively from 0 to 3. Immunostaining results were scored as the sum of the extent and intensity of immunoreactivity, considering a score ≥3 positive and a score <3 negative. Only cases with nuclear staining were considered positive for TTF1, p63, and p40, while those with cytoplasmic/membranous staining were considered positive for CK5/6 and CK7, and those with characteristic granular and cytoplasmic staining were considered positive for Napsin.

### Statistical analysis

IBM SPSS20 statistical package was used for data analyses unless otherwise specified. The relationship of clinical-pathological characteristics with marker expression was evaluated using the chi-square (χ^2^) or Fisher’s exact tests. Odds ratios with 95% confidence intervals (CIs) were calculated for 2 × 2 tables. Accuracy measures were calculated for each marker with the corresponding 95% CIs. *p* ≤ 0.05 was considered statistically significant. Survival analyses were performed with R 3.4.0 software, using the ‘survival’ (v2.42–3) and ‘survminer’ (v0.4.2) packages. Clinical information and normalized gene expression data were downloaded from Firebrowse (v1.1.38; http://firebrowse.org/) for the TCGA-LUSC project (*n* = 504). Patients with SCC from the TCGA-LUSC cohort were divided into two groups for analysis of the relationship between survival outcomes and gene expression: ‘high’ (above the median for the specific gene) and ‘low’ (below the median). Kaplan–Meier curves were plotted for the raw survival data, and Cox proportional hazards models were then constructed, accounting for tumour stage and for patient age and sex.

## Results

### Expression of conventional markers

We studied six well-documented markers conventionally considered of utility for the differential diagnosis of SCC and AC and routinely used by many histopathology laboratories (34–37). CK5/6, p40, and p63 are considered markers of SCC, while CK7, TTF1, and Napsin A are considered markers of AC. In our cohort of 85 patients, the most sensitive conventional marker for AC was CK7 (97.6%, with 2.4% false negatives), followed by Napsin A (80.5%, with 19.5% false negatives) and TTF1 (75%, with 25% false negatives). However, the most specific marker was TTF1 (97.7%), followed by Napsin A (93.2%), and CK7 (56.8%).

The most sensitive conventional marker for SCC was p63 (95.5%), followed by CK5/6 (93.2%) and p40 (88.6%), whereas p40 was the most specific (95.1%), followed by CK5/6 (92.7%) and p63 (85.4%). Other statistical analyses evaluating agreement and reproducibility are displayed in Tables 1 and 2. Among the conventional markers for AC (CK7, TTF1, and Napsin A) and SCC (CK5/6, p40, and p63) analyzed in our cohort of patients, only TTF1 in AC and p40 in SCC were specific in ≥94% of the samples, with ≥94% true positives.

**Table 1.** Conventional marker expression. Sensitivity, specificity, percentage of true positives and percentage of true negatives, accuracy, Youden’s *J* statistic, and positive and negative likelihood ratio values for staining with markers conventionally used for the differential diagnosis of adenocarcinoma (CK7, TTF1, and Napsin A) and squamous cell carcinoma (CK5/6, p40, and p63).

	Squamous cell carcinoma			Adenocarcinoma		
	CK5/6	p40	p63	CK7	TTF1	Napsin A
Sensitivity (%) (positive/total)^a^	93.2 (41/44)	88.6 (39/44)	95.5 (42/44)	97.6 (40/41)	75 (30/40)	80.5 (33/41)
Specificity (%) (positive/total)^b^	92.7 (38/41)	95.1 (39/41)	85.4 (35/41)	56.8 (25/44)	97.7 (43/44)	93.2 (41/44)
PTP (%) (positive/total)^c^	93.2 (41/44)	95.1 (39/41)	87.5 (42/48)	67.8 (40/59)	96.8 (30/31)	91.7 (33/36)
PTN (%) (positive/total)^d^	92.7 (38/41)	88.6 (39/44)	94.6 (35/37)	96.2 (25/26)	81.1 (43/53)	83.7 (41/49)
Accuracy (%) (positive/total)^e^	92.9 (79/85)	91.8 (78/85)	90.6 (77/85)	76.5 (65/85)	86.9 (73/84)	87.1 (74/85)
Youden’s *J* statistic^f^	0.85	0.84	0.81	0.54	0.73	0.74
LR+^g^	12.64	18.08	6.54	2.26	32.61	11.84
LR−^h^	0.08	0.12	0.05	0.04	0.26	0.21

a Sensitivity = TP/(TP + FN).

b Specificity = TN/(TN + FP).

c PTP = TP/(TP + FP).

d PTN = TN/(TN + FN).

e Accuracy = (TP + TN)/(TP + FP + FN + TN).

f Youden’s *J* statistic  =  Sensitivity + Specificity-1.

g Positive LR (LR+) = Sensitivity/(1-Specificity).

h Negative LR (LR-) = (1-Sensitivity)/Specificity.

FN: false negatives; FP: false positives; LR: likelihood ratio; PTN: percentage of true negatives; PTP: percentage of true positives; TN: true negatives; TP: true positives.

**Table 2.** Area under the curve (AUC) values for conventional and novel markers.

Marker	AUC	Asymptotic significance	95% Asymptotic confidence interval	
			Lower limit	Upper limit
PKP1_mb	0.861	0.000	0.773	0.950
DSG3_mb	0.813	0.000	0.712	0.913
KRT15_mb	0.861	0.000	0.773	0.949
TTF1	0.869	0.000	0.784	0.954
Napsin A	0.887	0.000	0.808	0.966
CK7	0.863	0.000	0.779	0.946
p63	0.965	0.000	0.923	1.000
CK5/6	0.960	0.000	0.914	1.000
p40	0.938	0.000	0.880	0.996

AUC: area under the curve.

### Expression of novel SCC markers

**Figure 1.**
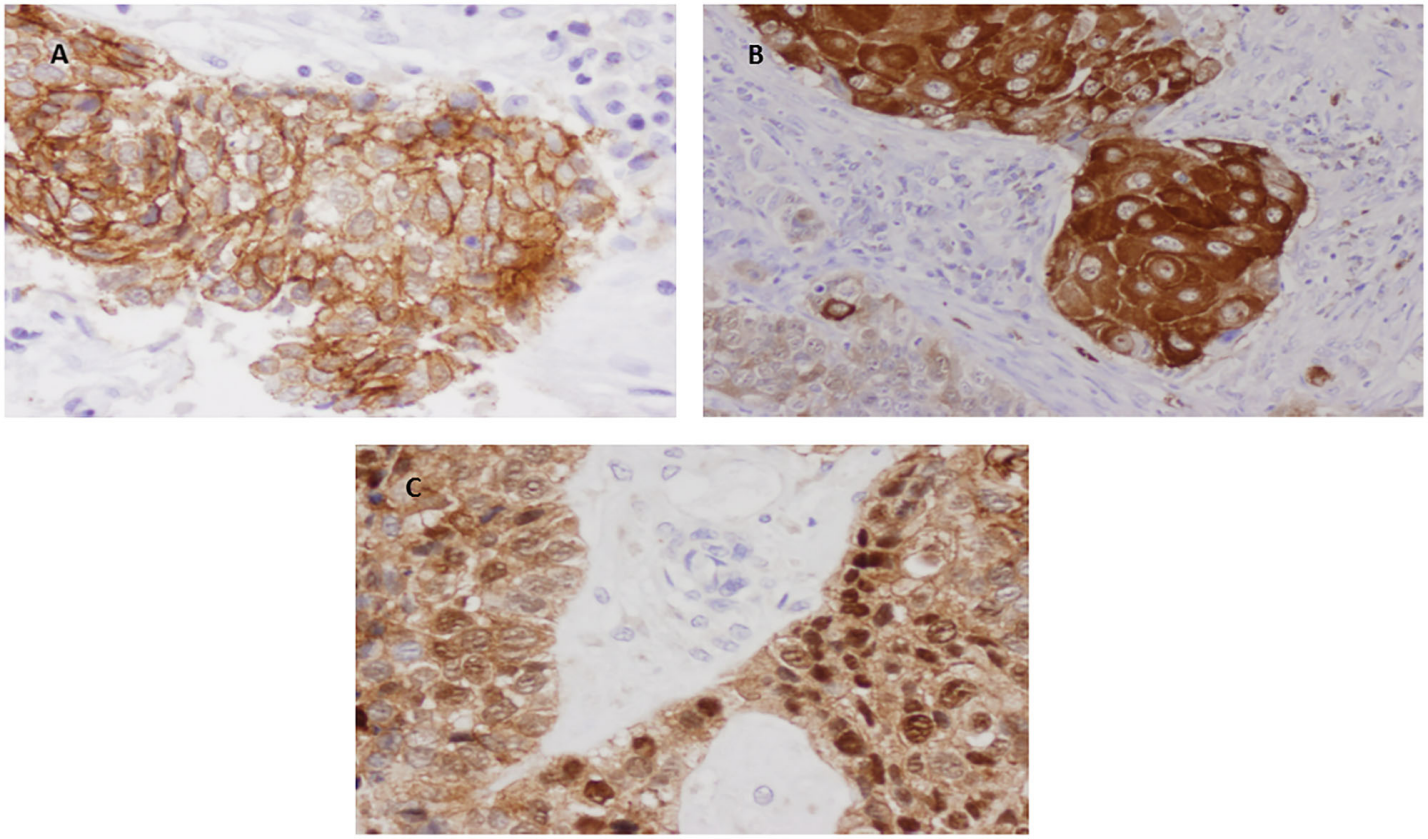
Immunohistochemical staining of PKP1 in SCC. (A) Predominant staining in cell membrane a (40×). (B) Positivity in membrane and cytoplasm. Note weak staining in less differentiated area (bottom left) (20×). (C) Nuclear staining (40×).

**Figure 2.**
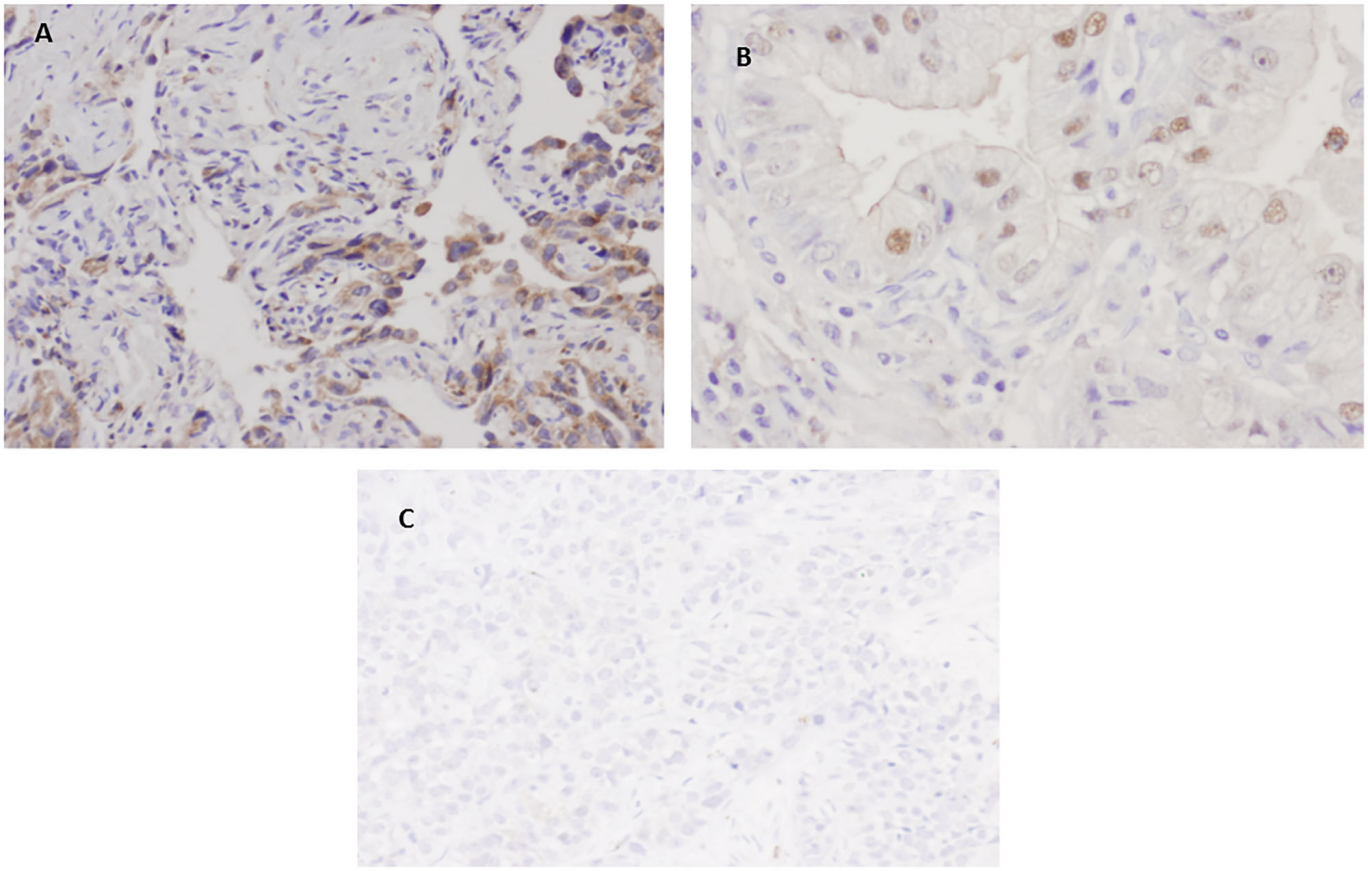
Immunohistochemical staining of PKP1 in AC. (A and B) Weak cytoplasmic and nuclear staining (40×). (C) Poorly differentiated AC with completely negative staining (10×).

**Figure 3.**
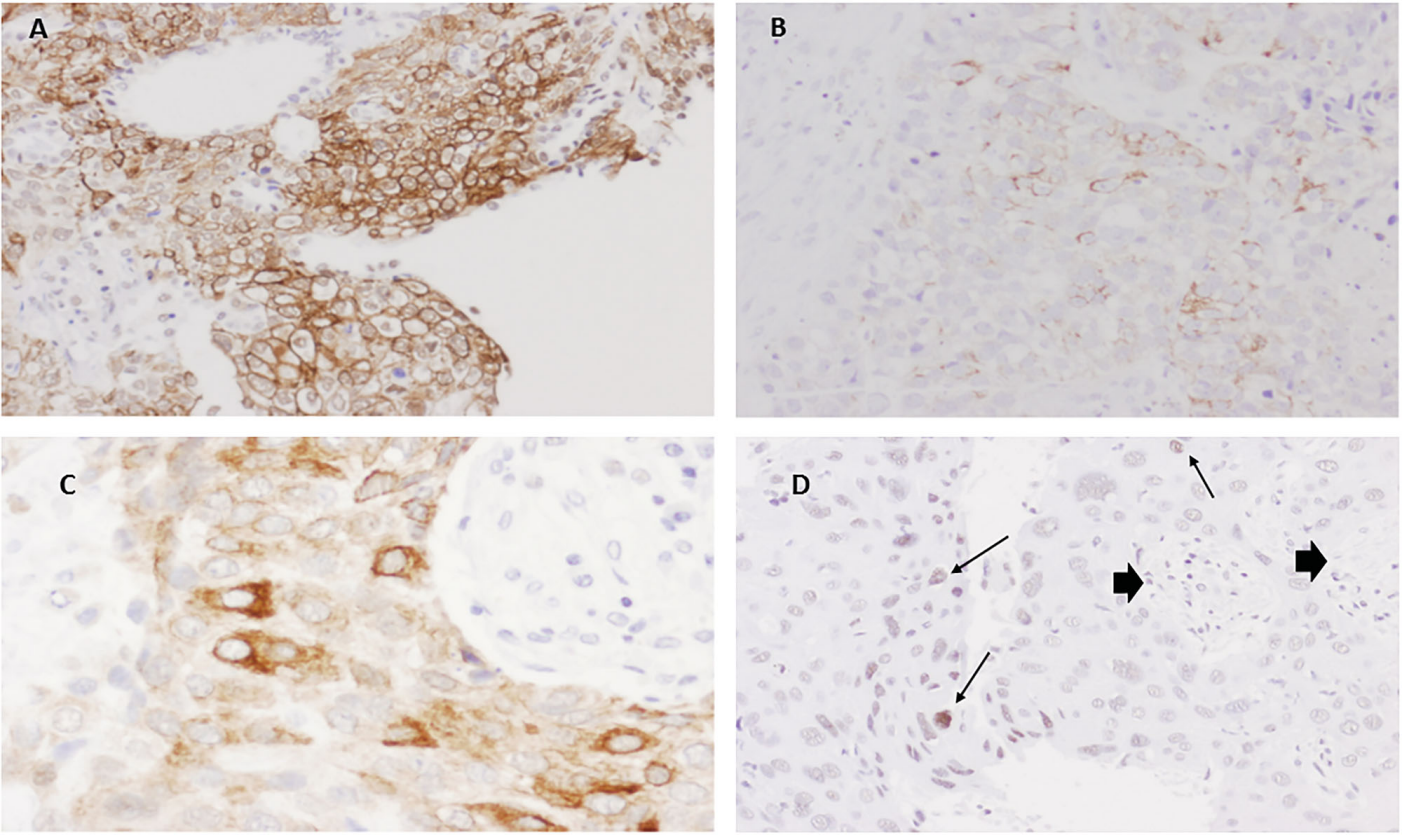
Immunohistochemistry for KRT15 in SCC. (A) Extensive positivity in well differentiated SCC in cell membranes (20×). (B) Poorly differentiated area with focal membranous staining (20×). (C) Area with predominant cytoplasmic staining (60×). (D) Poorly differentiated SCC showing some nuclei with faint staining (arrows). Note negativity in stromal cells (arrow heads) (20×).

We studied three novel markers for SCC (PKP1, KRT15, and DSG3) that may contribute to the differential diagnosis of SCC and AC, also analyzing their localization (nucleus, cytoplasm, or membrane). Immunohistochemical staining for PKP1 was mainly detected in SCC, with a heterogeneous distribution and intensity among the different tumours analyzed, and in different areas of the same neoplasm. PKP1 most frequently stained cellular membranes, marking intercellular junctions, followed by staining of the cytoplasm. Membranous staining was mainly observed in well to moderately differentiated areas of SCC. Nuclear staining was less often observed and was more frequent in cells with a more immature appearance (Figure 1). Membranous staining was never observed in ACs, although focal staining in nucleus and cytoplasm was occasionally observed (Figure 2). KRT15 was also mainly restricted to SCC (Figure 3), with variable distribution and intensity among different SCCs and among different areas of the same neoplasm. Staining in SCC was also observed in better-differentiated areas, mainly in cell membrane but also in cytoplasm. Nuclear staining was less frequently observed than with PKP1 and was seen in occasional cells with less-differentiated appearance (Figure 3). In ACs, KRT15 was absent in cell membrane and only occasionally present in a few cells localized in nucleus and cytoplasm.

**Figure 4.**
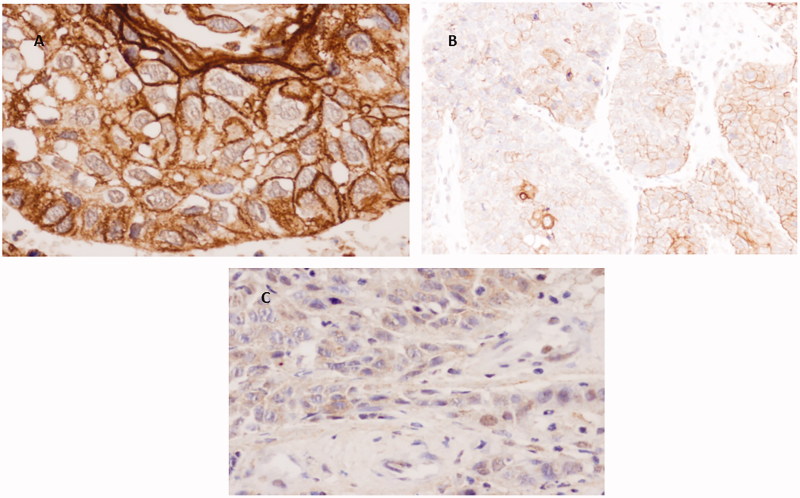
Immunohistochemical staining of DSG3 in SCC. (A) Well differentiated SCC with intense positivity mainly in cell membranes (40×). (B) More heterogeneous staining in cell membranes and some cytoplasms (20×). (C) Faint cytoplasmic/nuclear staining in poorly differentiated area (40×).

Staining for DSG3 was not observed in ACs, with the exception of only a few nuclei in a small number of cases. As occurred with PKP1 and KRT15, DSG3 stained SCCs with irregular distribution and was more often observed in cell membranes of better-differentiated areas and to a lesser degree in cytoplasms. Nuclear staining was much less frequent and mainly observed in poorly differentiated areas (Figure 4).

In our cohort, the most sensitive marker was PKP1 in nucleus (86%), followed by KRT15 in membrane (75.6%) and DSG3 in membrane (64.3%). However, DSG3 in membrane was the most specific (100%), followed by PKP1 in membrane (97.4%) and KRT15 in membrane (94.6%). Other statistical analyses evaluating agreement and reproducibility are displayed in Tables 2 and 3.

**Table 3.** Expression of novel SCC markers. Sensitivity, specificity, percentage of true positives and percentage of true negatives, accuracy, Youden’s *J* statistic, and positive and negative likelihood ratio values for PKP1, KRT15, and DSG3 staining according to their nuclear, cytoplasmic, or membranous localization.

	PKP1			KRT15			DSG3		
	Nucleus	Cytoplasm	Membrane	Nucleus	Cytoplasm	Membrane	Nucleus	Cytoplasm	Membrane
Sensitivity (%) (positive/total)^a^	72.1 (31/43)	86 (37/43)	76.7 (33/43)	19.5 (8/41)	73.2 (30/41)	75.6 (31/41)	16.7 (7/42)	61.9 (26/42)	64.3 (27/42)
Specificity (%) (positive/total)^b^	92.3 (36/39)	82.1 (32/39)	97.4 (38/39)	73 (27/37)	94.6 (35/37)	94.6 (35/37)	89.7 (35/39)	94.9 (37/39)	100 (39/39)
PTP (%) (positive/total)^c^	91.2 (31/34)	84.1 (37/44)	97.1 (33/34)	44.4 (8/18)	93.8 (30/32)	93.9 (31/33)	63.6 (7/11)	92.9 (26/28)	100 (27/27)
PTN (%) (positive/total)^d^	75 (36/48)	84.2 (32/38)	79.2 (38/48)	45 (27/60)	76.1 (35/46)	77.8 (35/45)	50 (35/70)	69.8 (37/53)	72.2 (39/54)
Accuracy (%) (positive/total)^e^	81.7 (67/82)	84.1 (69/82)	86.6 (71/82)	44.9 (35/78)	83.3 (65/78)	84.6 (66/78)	51.9 (42/81)	77.8 (63/81)	81.5 (66/81)
Youden’s *J* statistic^f^	0.64	0.68	0.74	−0.08	0.68	0.70	0.06	0.57	0.643
LR+^g^	9.36	4.80	29.50	0.72	13.56	14.00	1.62	12.14	–
LR−^h^	0.30	0.17	0.24	1.10	0.28	0.26	0.93	0.40	0.357

a Sensitivity = TP/(TP + FN).

b Specificity = TN/(TN + FP).

c PTP = TP/(TP + FP).

d PTN = TN/(TN + FN).

e Accuracy = (TP + TN)/(TP + FP + FN + TN).

f Youden’s *J* statistic  =  Sensitivity + Specificity-1.

g Positive LR (LR+) = Sensitivity/(1-Specificity).

h Negative LR (LR-) = (1-Sensitivity)/Specificity.

FN: false negatives; FP: false positives; LR: likelihood ratio; PTN: percentage of true negatives; PTP: percentage of true positives; TN: true negatives; TP: true positives.

According to these results, membranous staining with the novel markers (PKP1, KRT15, and DSG3) was specific for ≥94% of the SCC samples, obtaining ≥94% true positives in our cohort of patients.

The antibodies were also tested in combination. For SCC samples, membranous PKP1 staining showed a specificity of 97.4% and sensitivity of 76.7% and membranous DSG3 staining a specificity of 100% and sensitivity of 64.3% when separately evaluated. When considered together, membranous staining with these two markers achieved a specificity of 100% (95% CI = 90.6%–100.0%) but sensitivity of 52.4% (95% CI = 37.7%–66.6%). Positive TTF1 staining provided a specificity of 97.7% and a sensitivity of 75% for AC. When all three markers were considered together in Panel 1 SCC (positive membrane staining for PKP1 and DSG3 and negative staining for TTF1), the specificity for SCC samples was increased to 100.0% (95% CI = 90.6%–100.0%), but the sensitivity was 52.4% (95% CI = 37.7%–66.6%), with an AUC of 0.7619. Membranous KRT15 staining yielded a specificity of 97.4% and a sensitivity of 75.6% for SCC samples, and Napsin A staining a specificity of 93.2% and a sensitivity of 80.5% for AC samples when evaluated separately. When all five markers were considered together in Panel 2 SCC (positive membranous staining for PKP1, DSG3, and KRT15 and negative staining for TTF1 and Napsin A), the specificity for SCC samples was increased to 100.0% (95% CI = 89.6%–100.0%), but the sensitivity was 47.5% (95% CI = 32.9%–62.5%), with an AUC of 0.7375. For AC samples, the combined evaluation of negative membranous staining for PKP1 and DSG3 and positive TTF1, in Panel 1 AC, increased the specificity for AC samples to 100.0% (95% CI = 91.6%–100.0%), but the sensitivity was 24.3% (95% CI = 13.4%–40.1%), with an AUC of 0.8552. When negative membranous staining for PKP1, DSG3, and KRT15 and positive staining for TTF1 and Napsin A were considered together in Panel 2 AC, the specificity for AC samples increased to 100.0% (95% CI = 91.2%–100.0%), and the sensitivity was 63.6% (95% CI = 46.6–77.8%), with an AUC of 0.8088 (Table 4).

**Table 4.** Combination of antibodies useful for diagnosis of SCC and AC.

	Specificity	Sensitivity	AUC
Squamous cell carcinoma			
Panel 1 SCC: PKP1m+/DSG3m+/TTF1-	100%	52.4%	0.7619
Panel 2 SCC: PKP1m+/DSG3m+/KRT15m+/TTF1-/Napsin A-	100%	47.5%	0.7375
Adenocarcinoma			
Panel 1 AC: PKP1m-/DSG3-/TTF1+	100%	24.3%	0.8552
Panel 2 AC: PKP1m-/DSG3m-/KRT15m-/TTF1+/Napsin A+	100%	63.6%	0.8088

AUC: area under the curve.

### ROC curve analysis

The area under the ROC curve (or AUC) represents an optimal summary statistic for comparing the sensitivity and specificity of the nine markers (CK5/6, p40, p63, CK7, TTF1, Napsin A, PKP1, KRT15, and DSG3). For the cohort of 85 primary malignant lung neoplasms (41 ACs and 44 SCCs), including all histological grades, p63 (0.965) had the largest AUC, followed by CK5/6 (0.960), and p40 (0.938), with AUC values >0.9, while AUC values for the remaining markers were between 0.8 and 0.9. AUC values are displayed in Table 2.

### Stepwise multivariate logistic regression model

A stepwise multivariate logistic regression model was constructed to determine the optimal immunohistochemical marker panel for differentiating between SCC and AC. The best result included the combination of markers CK5/6, p63, and PKP1 in membrane, giving a percentage correct classification of 96.2% overall (94.6% of ACs and 97.6% of SCCs).

### Expression of novel SCC markers in relation to survival

**Figure 5.**
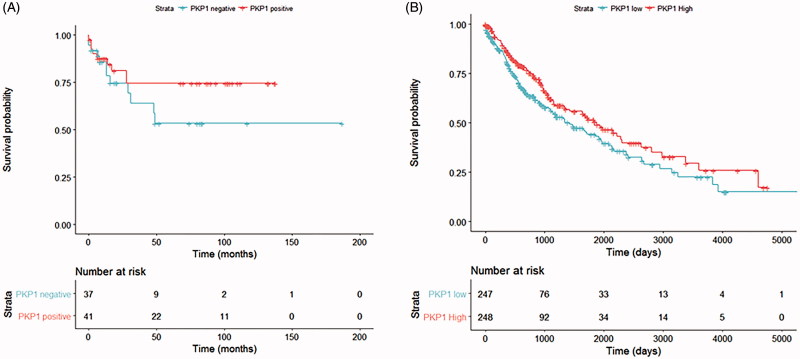
Survival analyses for PKP1 expression in lung cancer patients. (A) Survival analysis of our cohort of 38 SCC and 40 AC patients, classified as ‘PKP1-negative’ or ‘PKP1-positive’ according to the immunohistochemical staining of PKP1. (B) Survival analysis of the TCGA-LUSC cohort (*n* = 495 SCC patients), classified according to their RNA-Seq-measured PKP1 RNA level as ‘PKP1 low’ (below median value) or ‘PKP1 high’ (above median value).

Data on 80 patients were available for this analysis. The Kaplan–Meier survival curve depicted in Figure 5(A) shows a relationship between positive PKP1 staining and longer survival, although the difference was not statistically significant (log-rank *p* = 0.19), possibly due to the limited sample size. There was also a non-significant trend for longer survival in SCC versus AC patients (log-rank *p* = 0.47). In order to overcome this sample size limitation and remove tumour subtype as a confounder, we also studied 495 patients in the TCGA-LUSC cohort (The Cancer Genome Atlas) for whom survival and gene expression data were available (Supplemental Table 1, available online). In this cohort, a significant relationship was found between higher PKP1 expression and better overall survival (Figure 5(B)), obtaining a hazard ratio of 0.951 (95% CI = 0.907–0.997; *p* = 0.036) in a Cox proportional hazards model after adjusting for confounding variables.

We also performed survival analyses for all conventional and novel SCC biomarkers in the TCGA cohort under the same conditions as the PKP1 analysis. For the univariate analyses, we stratified the patients as ‘high’ or ‘low’ for each marker based on the expression being above or below the median value, and we plotted Kaplan–Meier curves (Supplemental Figures 1 and 2, available online). In the multivariate analyses, we applied Cox proportional hazard models for each marker, accounting for patient age, sex, and tumour stage, in order to assess the relationship of expression at mRNA level with patient survival.

In the multivariate analyses, the high expression of three SCC markers was associated with better survival: p63 (*p* = 0.007, HR = 0.93 [0.88–0.98]), PKP1 (*p* = 0.036, HR = 0.95 [0.91–1.0], as already noted), and CK5 (*p* = 0.018, HR = 0.95 [0.91–0.99]). In addition, the association of DSG3 with survival was close to statistical significance (*p* = 0.054, HR = 0.96 [0.93–1.0]). Expression of KRT15 or p40 at the mRNA level was not associated with patient survival.

## Discussion

In this study, we selected three novel markers (PKP1, KRT15, and DSG3) for a detailed evaluation and compared them with six conventional markers that are well described in the literature and are routinely used by many histopathology laboratories (34–37). Given that a very high proportion (up to 70%) of lung carcinomas are unresectable at their diagnosis (3), the biopsy sample is often the only available material for a correct subtyping of the neoplasm, and the present study focussed on small incisional biopsies taken during surgery. The precise subtyping of NSCLCs is essential to select the appropriate therapeutic approach. It is now recommended to complement the morphological criteria established by the WHO (2) with histochemical (e.g. mucin staining) and immunohistochemical techniques alongside molecular tests, whenever possible, especially for poorly differentiated tumours and for the analysis of small biopsy samples (38). In many histopathology laboratories, small biopsies are routinely subtyped using the combination of an AC marker (TTF1) and an SCC marker (p63 or p40) (38–40). However, there are several pitfalls in the differential diagnosis between SCC and AC by immunohistochemistry. For instance, the most widely used clones of TTF1 monoclonal antibodies are 8G7G3/1 and SPT24, which have been reported to have different sensitivities and specificities, with 8G7G3/1 being more specific and SPT24 more sensitive (41). Among SCC markers, p40 is considered the most specific but can be positive in 3% of ACs (42).

Studies in whole-tissue sections indicate that SCCs have a relatively precise immunophenotype, i.e. negativity for TTF1 and positivity for p63, CK5/6, and 34βE12. In contrast, ACs are much more heterogeneous, and only diffuse positivity for TTF1 is considered characteristic, given that a proportion of ACs also express markers considered typical of SCCs. Hence, albeit useful, no SCC marker is wholly specific (43).

According to the present results, among the expressions of the conventional markers CK7, TTF1, and Napsin A, characteristic of AC, and CK5/6, p40, and p63, characteristic of SCC, only TTF1 in AC and p40 in SCC were specific in ≥94% of samples in the present cohort, with ≥94% true positives. Membrane staining with the novel markers (PKP1, KRT15, and DSG3) was specific for ≥94% of the SCC samples, with ≥94% true positives in the present cohort. The antibody panel studied was most effective for the classification of SCC and AC when the antibodies were applied successively in a stepwise manner. We found that the specificity was higher (100%) when the antibodies were used in combination rather than individually. For SCC, the most effective panels were: Panel 1 SCC (positive staining of membrane for PKP1 and DSG3 and negative staining for TTF1; AUC of 0.7619) and Panel 2 SCC (positive staining of membrane for PKP1, DSG3, and KRT15 and negative staining for TTF1 and Napsin A; AUC of 0.7375). For AC, the most effective panels were: Panel 1 AC (negative staining of membrane for PKP1 and DSG3 and positive staining for TTF1; AUC of 0.8552) and Panel 2 AC (negative staining of membrane for PKP1, DSG3, and KRT15 and positive staining for TTF1 and Napsin A; AUC of 0.8088).

The novel markers described here showed a heterogeneous staining of SCC, which was observed in the membranes and cytoplasm of more differentiated cells, marking the intercellular junctions, with staining of nuclei more frequently detected in areas of more immature appearance. In AC samples, focal staining with these novel markers was detected in nucleus and cytoplasm, but never in membrane. In addition to their role in cell adhesion, plakophilins, including PKP1, have been reported to localize to the cytoplasm and nucleus, where they are thought to have several functions that are not completely understood (18). This explains the nuclear and cytoplasmic positivity observed, mainly in SCC in our series. In a few cases and in a few cells, we have also seen occasional nuclear staining for CKT15 and DSG3. Although nuclear localization of CKT15 and DSG3 has not been reported (22,23) and non-specific staining cannot be ruled out, it has been shown that several cytoskeletal proteins, formerly thought to be exclusively cytoplasmic, and including some keratins (keratins 7, 8, 17, and 18), are components of the nuclear matrix, where they may have multiple functions. Some studies of skin and cervical tumours indicated that keratin 17 has a role in the cell cycle and in gene expression regulation (44). These data and the fact that KRT15 and DSG3 are not routinely used in most laboratories prompted our assessment of nuclear staining. Further investigation is warranted to explore the significance of our findings.

In our cohort of patients, the relationship of PKP1 staining with better survival of SCC and AC patients did not reach statistical significance; however, we were able to confirm the relationship between high PKP1 mRNA expression and better overall survival in an additional analysis of 495 SCC patients from TCGA. In addition, our TCGA analysis revealed a close-to-significant association between high DSG3 mRNA expression and improved overall survival. This agrees with previous reports that associated positive DSG3 staining with longer survival in lung cancer patients of all histologic subtypes (37). Taken together, the results indicate a relationship of high PKP1 (and, to a lesser extent, DSG3) RNA and protein levels with longer overall survival. Hence, PKP1 and DSG3 expression levels not only serve as specific markers for SCC but may also have potential prognostic value.

According to our findings, evaluation of CK15 and especially of DSG3 and PKP1 improves the differential diagnosis of SCC and AC. However, a potential limitation of these SCC markers is that membrane-specific positivity is found in better-differentiated samples that are more readily identifiable under the microscope. Nevertheless, they may be especially useful in samples affected by a major artefact, because they offer a more objective parameter for evaluating the degree of differentiation. A further advantage of these markers is the complete absence of staining in poorly differentiated ACs, observing cytoplasmic and nuclear positivity only in moderately or well-differentiated ACs. These antibodies could possibly be used in a cocktail, with the consequent saving of histological sections. This is an important issue, given the need for genetic as well as immunohistochemical analyses in tissue from small biopsies in patients with lung carcinoma.

Despite the small number of samples analyzed, the stepwise multivariate logistic regression model showed that the combination of markers CK5/6, p63, and PKP1 in membrane gave a percentage correct classification of 96.2% overall (94.6% of AC and 97.6% of SCC), being the best immunohistochemical marker panel to distinguish between SCC and AC.
